# Genomic analysis of the Streptococcus pneumoniae in Taiwan: a nationwide study from 2006 to 2022

**DOI:** 10.1099/mgen.0.001498

**Published:** 2025-09-09

**Authors:** Hsueh-Chien Raymond Cheng, Shu-Chen Kuo, Jui-Fen Lai, Han-Chieh Wu, Feng-Jui Chen, Huey-Kang Sytwu, Stephanie W. Lo, Stephen D. Bentley, Ying-Chi Huang

**Affiliations:** 1 Wellcome Trust Sanger Institute, Wellcome Trust Genome Campus, Hinxton, Cambridge, UK; 2 National Institute of Infectious Diseases and Vaccinology, National Health Research Institutes, Zhunan, Taiwan, ROC; 3 Milner Centre for Evolution, Department of Life Sciences, University of Bath, Bath, UK; 4 Institute of Epidemiology and Preventive Medicine, College of Public Health, National Taiwan University, Taipei, Taiwan, ROC

**Keywords:** Global Pneumococcal Sequence Cluster (GPSC), invasive pneumococcal disease (IPD), *Streptococcus pneumoniae*, Taiwan, whole-genome sequencing

## Abstract

*Streptococcus pneumoniae* remains a leading respiratory pathogen for children and the elderly. In Taiwan, a national PCV13 catch-up vaccination programme for children began in March 2013. This study investigates the population structure and antimicrobial profiles of pneumococcal isolates in Taiwan from 2006 to 2022. A total of 1,343 *S*. *pneumoniae* isolates were collected biennially from 27 hospitals across 4 regions of Taiwan. All isolates were analysed for serotypes and antimicrobial resistance (AMR) profiles, and 137 isolates causing invasive pneumococcal diseases (IPDs) underwent whole-genome sequencing. In the post-PCV13 era (2014–2022), serotypes 15A, 23A, 19A, 19F and 6A predominated. The elderly populations showed an increased proportion of IPD in the post-PCV13 era. Among the 137 IPD isolates, GPSC1 (21.9%) was the primary lineage, consistently harbouring AMR genes. Serotype 15A within GPSC9 emerged rapidly, particularly in central Taiwan. In GPSC16, non-PCV13 serotype 15B/C increased significantly, replacing vaccine types. GPSC6 displayed frequent capsular switching, a unique phenomenon in Taiwan, driven by distinct recombination events post-2000. These findings underscore the importance of sustained genomic surveillance of *S. pneumoniae* in Taiwan to monitor age-related shifts in IPD burden and to track the expansion of persistent and highly adaptable lineages such as GPSC1 and GPSC6.

**Impact Statement**

This study presents the first nationwide, longitudinal whole-genome sequencing analysis of *Streptococcus pneumoniae* in Taiwan, integrating 16 years of surveillance data from the Taiwan Surveillance of Antimicrobial Resistance (TSAR) programme. By combining genomic and epidemiological data, we uncover the evolutionary trajectories of invasive pneumococcal lineages under vaccine and antibiotic selection pressures. Taiwan’s unique setting, characterized by dense population, high vaccine coverage, widespread antibiotic use and substantial population mobility, has shaped a distinct pneumococcal landscape marked by both local adaptation and international strain introduction. Notably, we identify lineage-specific recombination patterns, including frequent capsular switching within GPSC6 and the persistent expansion of multidrug-resistant GPSC1/19A. Our findings underscore the critical role of long-term genomic surveillance in detecting emerging threats, guiding vaccine policy and informing antimicrobial resistance control strategies in the post-PCV era.

## Data Summary

Whole-genome sequences are deposited at the European Nucleotide Archive (ENA), and the accession numbers are included in Table S1 (available in the online Supplementary Material 1). All supplementary tables and figures are available online (Supplementary Material 1). A phylogenetic snapshot of pneumococcal isolates from Taiwan is also available at this link: https://microreact.org/project/rzJGdFK6MBWANcfLy1K3rH-taiwan-137-isolates. The authors confirm that all supporting data, code and protocols have been provided within the article or through supplementary data files.

## Introduction

*Streptococcus pneumoniae* (the pneumococcus) is the leading cause of pneumonia, sepsis and meningitis in humans [1,2]. The pneumococcus can cause invasive pneumococcal diseases (IPDs) when it invades normally sterile sites, leading to morbidity and mortality among young children and the elderly and contributing substantially to the infectious disease burden in these age groups [2,3]. Pneumococcal conjugate vaccines (PCVs) targeting the capsule surrounding pneumococcal cells are effective in reducing pneumococcal disease [2].

In Taiwan, a government-funded PCV13 catch-up vaccination programme was launched in March 2013 for children aged 2–5 years and expanded to include those aged 1–5 years in 2014 [4]. In 2015, PCV13 was incorporated into the national infant immunization schedule, achieving over 80% coverage. The PCV13 vaccine targets serotypes 1, 3, 4, 5, 6A, 6B, 7F, 9V, 14, 18C, 19A, 19F and 23F. For adults aged 65 years and older, the 23-valent pneumococcal polysaccharide vaccine (PPSV23) was gradually implemented starting in 2007 and covers all PCV13 serotypes plus additional ones, including 2, 8, 9N, 10A, 11A, 12F, 15B, 17F, 20 and 22F [5]. While these vaccination strategies have significantly reduced the burden of PCV13 vaccine-type (VT) disease, the prevalence of non-vaccine serotypes (NVTs) and vaccine escape variants has increased in the post-PCV13 era, many of which are associated with multidrug resistance (MDR) [6,7].

The Taiwan Surveillance of Antimicrobial Resistance (TSAR), established in 1998, is a nationwide programme that systematically collects *S. pneumoniae* isolates from patients of all ages across more than 20 hospitals in 4 geographic regions. A previous TSAR study using serotyping and multilocus sequence typing (MLST), which characterized seven housekeeping genes that are conserved across strains, documented substantial shifts in serotype distribution after PCV13 catch-up programme, notably the emergence of multidrug-resistant NVTs such as 15A and 23A [6]. However, traditional methods such as serotyping and MLST have limited resolution for delineating prevalent lineages, particularly in identifying pneumococcal strains that undergo capsular switching within the same genetic background. This phenomenon is often driven by vaccine-induced selective pressure and occurs through recombination at the capsular polysaccharide synthesis (*cps*) locus [7]. Whole-genome sequencing (WGS) overcomes these limitations by enabling detailed analysis of recombination at the *cps* locus, precise lineage assignment and comprehensive tracking of resistance determinants, thereby providing critical insights into serotype replacement, clonal expansion and pneumococcal evolution in the post-PCV era [7].

In this study, we conducted the first nationwide genomic study of *S. pneumoniae* in Taiwan spanning both the pre- and post-PCV13 eras (2006–2022). The study consisted of two components: (1) age-stratified serotype analysis across children, adults and the elderly, including both invasive and non-invasive isolates, to assess temporal and age-specific trends, and (2) WGS of IPD isolates to elucidate lineage structure and antimicrobial resistance mechanisms. We applied the Global Pneumococcal Sequence Cluster (GPSC) framework, which incorporates core and accessory genome variation for standardized lineage assignment and enables tracking of clonal replacement and capsular switching events [8,9]. These findings provide critical evidence to inform future pneumococcal vaccine strategies and antimicrobial stewardship policies in Taiwan.

## Methods

### Isolate collection protocol and identification

The TSAR programme conducted biennial surveys from July to September using a standardized two-stage isolate collection strategy. In the first stage (regular collection), each hospital was required to consecutively collect and submit 200 clinical isolates regardless of bacterial species or specimen type. These included 50 isolates from outpatients, 30 from adult intensive care unit (ICU) patients, 100 from adult non-ICU inpatients and 20 from paediatric patients. In addition, 20 isolates (in 2006) or 50 isolates (from 2008 to 2022) were collected from blood or other sterile body sites, irrespective of species. In the second stage (special collection), all *S. pneumoniae* isolates were submitted during the 3-month survey period, regardless of specimen type, to support pneumococcal epidemiological analysis.

Between 2006 and 2022, a total of 1,343* S. pneumoniae* isolates were collected. The source distribution of these isolates is summarized in Table S2. Among the 138 isolates initially identified as causing IPD, one was excluded after WGS-based species confirmation due to misidentification. The final dataset therefore included 137 confirmed IPD isolates subjected to WGS.

All isolates were stored at −70 °C. Species identification for isolates before 2018 followed the previously described protocols [6]. In brief, the identification of *S. pneumoniae* was confirmed based on colony morphology, *α*-haemolysis, optochin sensitivity and/or bile solubility tests [6]. The reagents and media were obtained from BBL (Becton Dickinson Microbiology System, Sparks, MD). After 2018, the isolates were identified by MALDI-TOF (Bruker Daltonik, Germany). The isolates were recovered from clinical samples taken as part of standard care. The TSAR project was approved by the Research Ethics Committee of National Health Research Institutes (NHRI), Taiwan (EC960205-E, EC1010602-E, EC1030406-E and EC1050606-E).

### Antimicrobial susceptibility test

Minimum inhibitory concentrations (MICs) of various antimicrobials were determined by broth microdilution (BMD) using Sensititre standard panels (Trek Diagnostics, Thermo Fisher Scientific, UK). The current Clinical and Laboratory Standards Institute (CLSI) breakpoints were applied [10]. Antimicrobial agents tested included *β*-lactams (amoxicillin/clavulanate, penicillin, ceftriaxone and meropenem) and non-*β*-lactams (clindamycin, erythromycin, levofloxacin, tetracycline, trimethoprim/sulfamethoxazole and vancomycin) across all study years, except for amoxicillin/clavulanate in 2008 and clindamycin in 2006, which were not tested. MDR was defined as resistance to ≥3 classes of antimicrobials from the following list: *β*-lactams (penicillin MIC ≥2 mg l^−1^), macrolides, lincosamides, tetracyclines, fluoroquinolones and folate pathway inhibitors [11].

### Serotyping by Quellung reaction and multiplex PCR

The serotype of pneumococcal isolates was determined by the Quellung reaction using commercial omni, pooled group and selected type antisera (Statens Serum Institut, Copenhagen, Denmark) on all isolates [12]. A multiplex PCR serotyping approach has also been used on all isolates since 2010 [13]. Isolates before 2010 that were omni-serum-positive but pooled group sera-negative were also subjected to PCR serotyping. Nontypeable (uncapsulated) isolates referred to those tested negative by omni-serum and multiplex PCR serotyping but positive for autolysin (*lytA*) gene by PCR [14].

### Sample preparation, DNA extraction and WGS

The 138 bacterial samples were grown up overnight, and single colonies were then collected for the following DNA extraction. Extraction of pneumococcal DNA was performed by using a QIAamp DNA Mini Kit (QIAGEN). WGS was performed using the Illumina HiSeq platform to produce paired-end reads of 151 bp in length.

WGS analysis was performed by the GPS pipeline (https://github.com/sanger-bentley-group/gps-pipeline). Briefly, the GPS pipeline takes the paired-end reads of each isolate as the input and carries out quality control (QC), *de novo* genome assembly, *in silico* MLST, GPSC typing, *in silico* serotyping and AMR prediction. For read QC, the total number of bases in sequence reads should be larger than 38 Mb. For taxonomy QC, we assigned taxonomic labels to sequencing reads by Kraken2, and the percentage of *S. pneumoniae* genomic content should be larger than 60%, while the genomic content from other genera should be less than 2% [15]. Shovill (https://github.com/tseemann/shovill) was used for *de novo* genome assembly, and QC of genome assembly was performed by QUAST where the assembly length for each isolate should range between 1.9 and 2.3 Mb with less than 500 contigs and larger than 20× coverage [16]. As a result, 137 of the 138 isolates passed the QC steps. After QCs, serotypes and sequence types were inferred from the genome assembly using SeroBA v1.0.7 and MLST v2.23 [17] (https://github.com/tseemann/mlst). GPSC lineage was designated by PopPUNK which considers the whole-genome content including both core and accessory genes using GPSC reference database v9 [9]. For AMR interpretation, CLSI guidelines were applied to classify the isolates into susceptible (S), intermediate (I) and resistant (R). More stringent meningitis cutoffs were applied to penicillin, cefotaxime and ceftriaxone resistance based on the CLSI guideline [10]. AMR prediction of six *β*-lactam antibiotics including penicillin, cefuroxime, ceftriaxone, cefotaxime, amoxicillin/clavulanate and meropenem was predicted from the genomic data using a machine learning model developed by the US CDC based on the allele types of penicillin-binding protein (PBP) 1A, 2B and 2X [18]. Other AMR prediction of 13 antibiotics was inferred by known genetic determinants, including chloramphenicol, clindamycin, trimethoprim/sulfamethoxazole, doxycycline, erythromycin, kanamycin, levofloxacin, rifampin, sulfamethoxazole, tetracycline, trimethoprim and vancomycin. The results of the WGS analysis are shown in Table S1.

### Phylogenetic analysis

The phylogeny of the 137 isolates was built to investigate the genetic relationship among the isolates. First, variant calling was performed by SKA2 v2 by mapping to a reference genome of *S. pneumoniae* ATCC 700669 [19]. A maximum-likelihood tree was then inferred by using FastTree [20]. To generate a GPSC6-specific and GPSC16-specific tree, we collected data from the Global Pneumococcal Sequencing Database (https://data-viewer.monocle.sanger.ac.uk/project/gps, GPS release 5.0). We first generated a reference-guided GPSC-specific reference genome by using ABACAS v1.3.1 to order the contigs relative to strain ATCC 700669 [21]. Any contigs which did not align were concatenated to the end. We aligned the isolates to the GPSC reference by BWA v0.7.17 alignment, and variants were called by SAMtools v1.19 [22,23]. We masked recombination sites using Gubbins v3.2.1 with RAxML and a general-time-reversible model to construct a recombination-free tree [24]. To investigate the time when the GPSC isolates were introduced in Taiwan and other countries and when the capsular switching event happened, we generated a time-resolved phylogeny for GPSC6 and GPSC16 isolates that rescaled the branch lengths to time by BactDating with an additive uncorrelated relaxed clock model [25].

### Statistical analysis

Statistical analyses were performed to compare the distribution of serotypes, GPSC and AMR between the pre-PCV13 period (2006–2012) and the post-PCV13 period (2014–2022) using Fisher’s exact test. Two-sided *P*-values <0.05 were considered statistically significant. *In silico* serotyping results were used for serotype comparisons. Serotypes were classified as VTs, comprising those included in PCV13, and NVTs, referring to serotypes not covered by the PCV13 vaccine. All statistical tests were performed in R v4.3.2.

## Results

### Serotype distribution and vaccine coverage trends (2006–2022)

A total of 1,343 *S*. *pneumoniae* isolates were collected through the TSAR programme from 2006 to 2022 biennially, with 10.2% (*n*=137) IPD isolates recovered from sterile anatomical sites. The 137 IPD isolates were mainly collected from blood (*n*=119, 86.9%), 9 isolates from pleural fluid, 5 isolates from cerebrospinal fluid, 3 isolates from ascites and 1 from pus obtained from a deep wound. Of the remaining 1,206 non-invasive isolates, the majority (83.2%) were collected from respiratory tract samples, including sputum and nasopharyngeal discharge. An additional 12.4% were collected from pus, 1.5% from ear discharge and 3% from unknown sources.

**Fig. 1.**
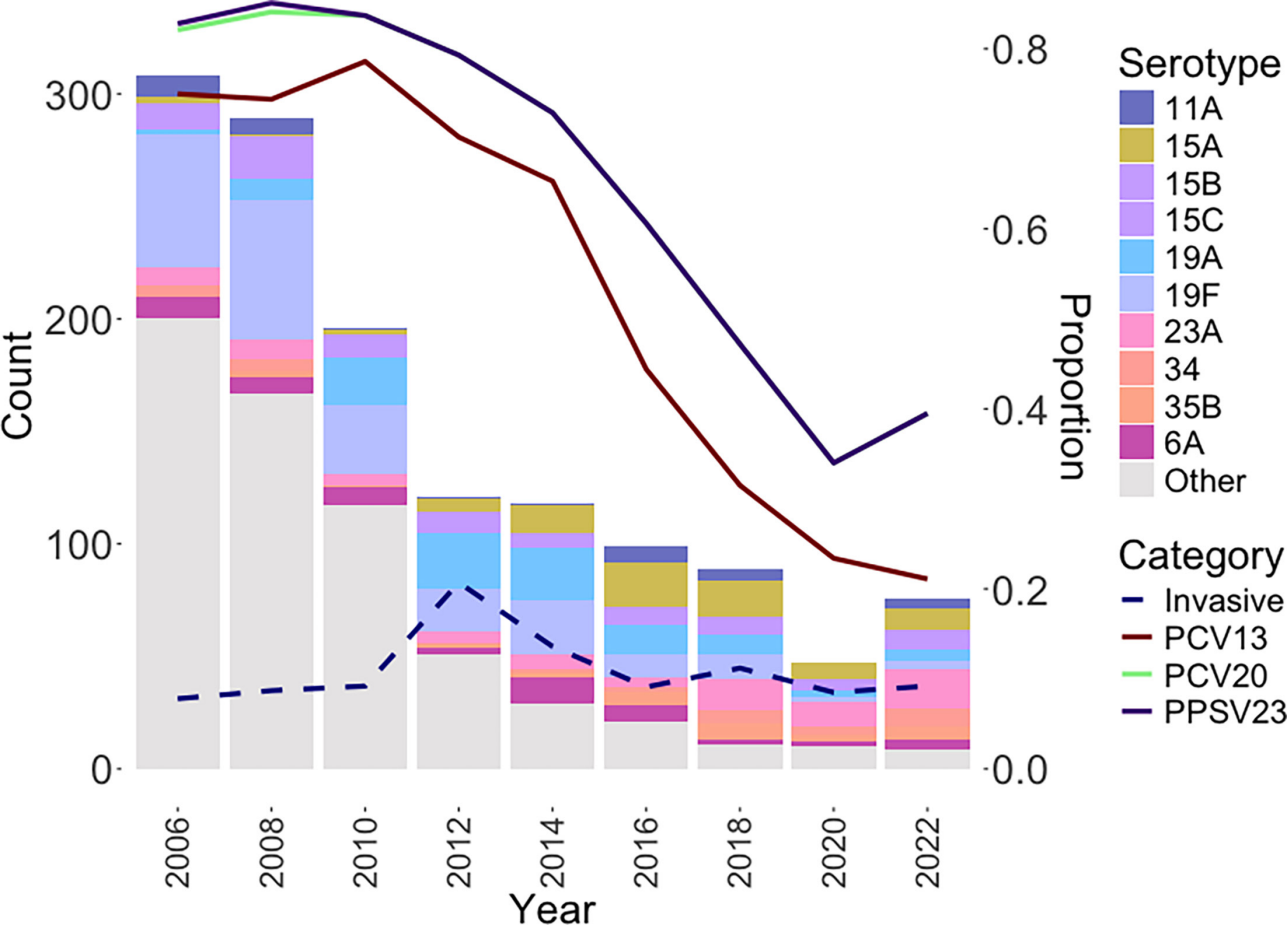
Serotype distribution of 1,343 pneumococcal isolates collected by the TSAR programme from 2006 to 2022. Bar charts display the annual counts of the most frequent serotypes (left y-axis). Line charts depict the yearly frequencies of vaccine-covered serotypes (PCV13, PCV20 and PPSV23, right y-axis). The dotted line chart represents the annual frequency of invasive isolates (right y-axis).

In the pre-PCV13 era (2006–2012), 914 (68.1%) isolates were collected, and 429 were collected during the post-PCV13 period (2014–2022). Following the catch-up programme of PCV13, the most prevalent serotypes were 15A (14.9%), 23A (12.6%), 19A (12.4%), 19F (11.9%), 6A (6.3%), 35B (5.6%), 34(4.9%), 15B (4.7%), 11A (4.2%) and 15C (4%), while other serotypes accounted for 18.6% of the isolates. The frequency of PCV13 serotypes gradually decreased from 75% in 2006 to 21.1% in 2022. In 2022, the frequency of PCV20 serotypes (comprising PCV13 serotypes plus 8, 10A, 11A, 12F, 15B and 22F) and PPSV23 serotypes was both 39.5% (Fig. 1 and Table S2). The proportion of invasive cases was 7.8% in 2006, peaking at 20.7% in 2012, before decreasing to 9.2% by 2022. The MDR rate was over 90% before 2014 and then decreased to 72.3% and 73.7% in 2020 and 2022, respectively (Table S2).

### Age-stratified serotype dynamics before and after PCV13 implementation

**Fig. 2.**
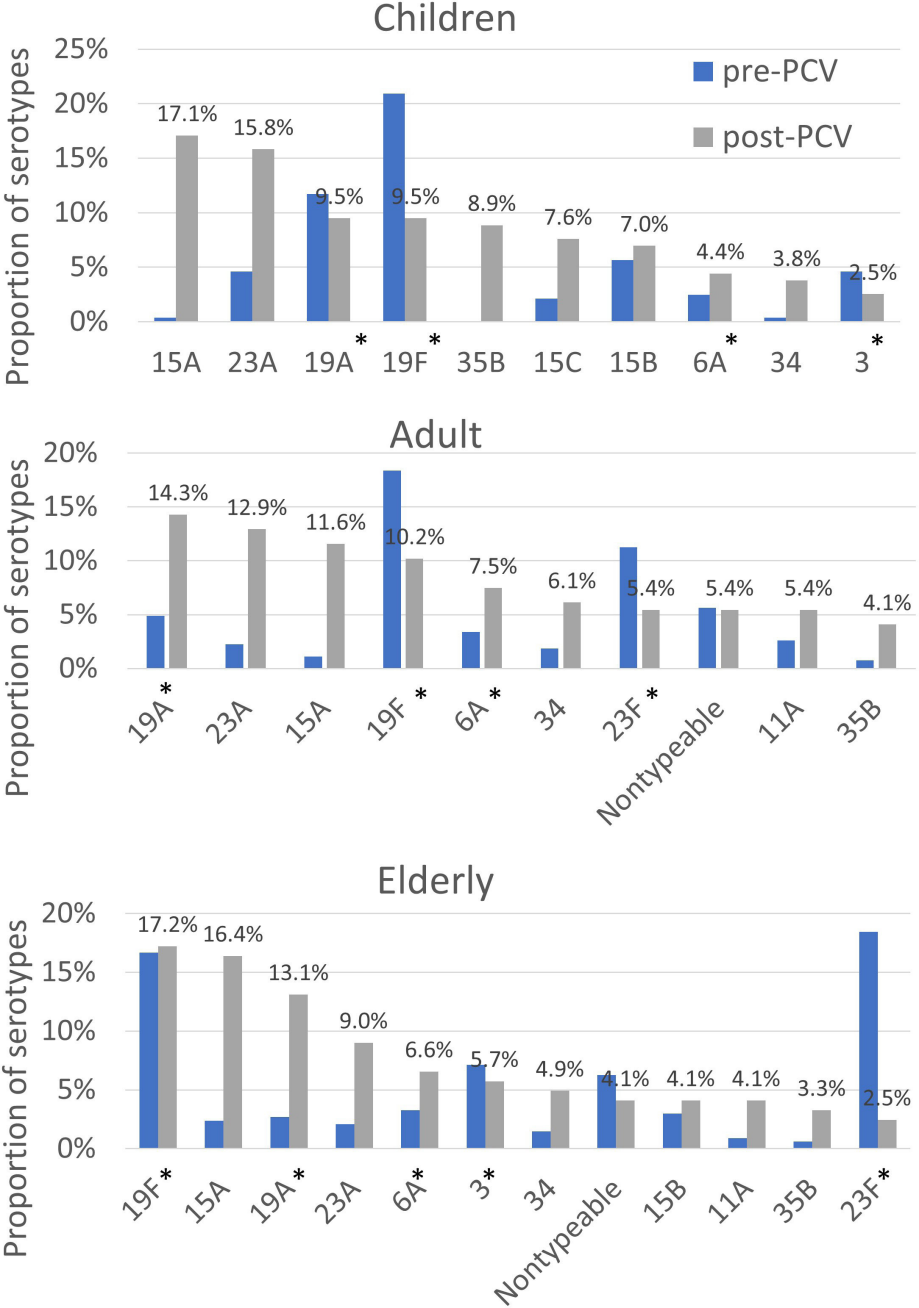
Serotypes of 1,343 pneumococcal isolates from the TSAR programme among children, adult and elderly groups from 2006 to 2022. Bar charts compare the proportion of the most common serotypes during the pre-PCV13 (2006–2012; blue) and post-PCV13 (2014–2022; grey) periods. Serotypes are ranked by their frequency in each age group during the post-PCV13 period. Percentages indicate the proportion of each serotype in the post-PCV13 period. *Serotypes covered by PCV13.

Fig. 2 presents the distribution of serotypes across children, adults and the elderly during the pre-PCV (2006–2012) and post-PCV (2014–2022) periods. Following the introduction of PCV13, the proportion of NVTs increased significantly in all age groups (all *P*<0.0001), indicating a substantial shift in serotype composition within the pneumococcal population in Taiwan.

In children, the proportions of VTs 19A and 19F declined markedly from 11.7% and 20.9% in the pre-PCV period to 9.5% each in the post-PCV period. Conversely, the NVTs 15A and 23A became predominant in the post-PCV period, accounting for 17.1% and 15.8% of isolates, respectively. Among adults, 19F decreased markedly from 18.4% to 10.2% after PCV13 introduction, while NVTs such as 23A (12.9%) and 15A (11.6%) became major contributors to the post-PCV13 disease burden. Notably, 19A, despite being vaccine-covered, remained the most common serotype (14.3%). In the elderly, 19F (a VT) remained the most prevalent serotype (17.2%), followed by NVT 15A (16.4%) and VT 19A (13.1%), with NVT 23A ranking fourth (9.0%). The persistence of both VT and NVT serotypes in this age group reflects incomplete vaccine impact and continued circulation of diverse lineages. These findings prompted further investigation into the clonal composition and genomic features underlying these serotype shifts.

Consistent with these trends, the proportion of NVTs 15A and 23A among IPD cases increased significantly in the post-PCV13 period, by 8.7% and 7.6%, respectively (*P*=0.042 and 0.043; Table S3). Serotype 15A emerged predominantly in elderly patients, whereas 23A was more frequently observed in adults (Table S4).

### Comparison of invasive and non-invasive pneumococcal isolates

We next compared the demographic and serotype characteristics of invasive and non-invasive isolates to evaluate their association with disease severity. Table 1 presents the characteristics of the 137 invasive isolates and compares them to those of 1,206 non-invasive *S. pneumoniae* isolates. The overall percentages of isolates responsible for IPD remained similar before and after the introduction of PCV13, at 10% and 10.7%, respectively, of all *S. pneumoniae* isolates. Among these 137 invasive isolates, 27.1% were collected from children, 40.1% from adults, 29.2% from elderly patients and the remaining 3.6% with unknown age (Table 1). However, the proportion of invasive ones among all pneumococci increased markedly in the elderly, from 2.5% in 2010 to 22.7% in 2022, while it declined in both paediatric and adult patients (Fig. S1).

**Table 1.** Comparison between invasive and non-invasive pneumococci in Taiwan, 2006–2022

	Invasive (*n*=137)	Non-invasive (*n*=1,206)	*P*-value
Period			0.665
Pre-PCV (2006–2012)	91 (66.4)	823 (68.2)	
Post-PCV (2014–2022)	46 (33.6)	383 (31.8)	
Location			0.255
ICU	22 (16)	209 (17.4)	
Non-ICU	54 (39.4)	561 (46.5)	
Outpatient (including ER)	59 (43.1)	426 (35.3)	
Unknown	2 (1.5)	10 (0.8)	
Region			0.07
Central	52 (38)	557 (46.2)	
Eastern	7 (5.1)	43 (3.6)	
Northern	38 (27.7)	358 (29.7)	
Southern	40 (29.2)	248 (20.5)	
Serotype*			
**19A**	21 (15.3)	89 (7.4)	0.001
**3**	15 (10.9)	70 (5.8)	0.019
**14**	13 (9.5)	61 (5.1)	0.031
**19F**	12 (8.8)	210 (17.4)	0.001
**23F**	12 (8.8)	133 (11)	0.417
**6B**	12 (8.8)	122 (10.1)	0.616
**6A**	8 (5.8)	47 (3.9)	0.277
15A	7 (5.1)	69 (5.7)	0.769
15B	6 (4.4)	51 (4.2)	0.934
23A	5 (3.6)	76 (6.3)	0.217
Other	26 (19)	278 (23.1)	
Hospital type			0.605
Medical centre	76 (55.5)	641 (53.2)	
Regional hospital	61 (44.5)	565 (46.8)	
Age group			0.130
Children	37 (27.1)	403 (33.4)	
Adult	55 (40.1)	359 (29.8)	
Elderly	40 (29.2)	416 (34.5)	
Unknown	5 (3.6)	28 (2.3)	
Antimicrobial non-susceptibility†			
Penicillin	45 (32.8)	343 (28.4)	0.281
Amoxicillin/clavulanate	28 (24.8)	235 (25)	0.577
Cefotaxime	30 (21.9)	225 (18.7)	0.359
Meropenem	77 (56.2)	711 (59)	0.536
Levofloxacin	6 (4.4)	82 (6.8)	0.278
Tetracycline	126 (92)	1,103 (91.5)	0.839
Erythromycin	125 (91.2)	1,127 (93.4)	0.33
Chloramphenicol	49 (35.8)	366 (30.3)	0.193
Trimethoprim/sulfamethoxazole	92 (67.2)	821 (68.1)	0.826

*Top ten serotypes among invasive pneumococci were listed here.

†Antimicrobial susceptibility rates were calculated by the results of microdilution here.

Serotypes in bold indicate vaccine serotypes covered by PCV13.

The most common serotypes of invasive infections were 19A (15.3%), 3 (10.9%), 14 (9.5%), 19F (8.8%), 23F (8.8%), 6B (8.8%), 6A (5.8%), 15A (5.1%), 15B (4.4%) and 23A (3.6%), collectively accounting for 81% of cases (Table 1). Notably, serotypes 19A (15.3% vs. 7.4%, *P*=0.001), 3 (10.9% vs. 5.8%, *P*=0.019) and 14 (9.5% vs. 5.1%, *P*=0.031) were significantly more common among invasive isolates compared with non-invasive ones, while 19F (8.8% vs. 17.4%, *P*=0.001) was significantly less frequent among invasive cases.

### Genomic characteristics of invasive pneumococci (GPSC, AMR and virulence)

**Fig. 3.**
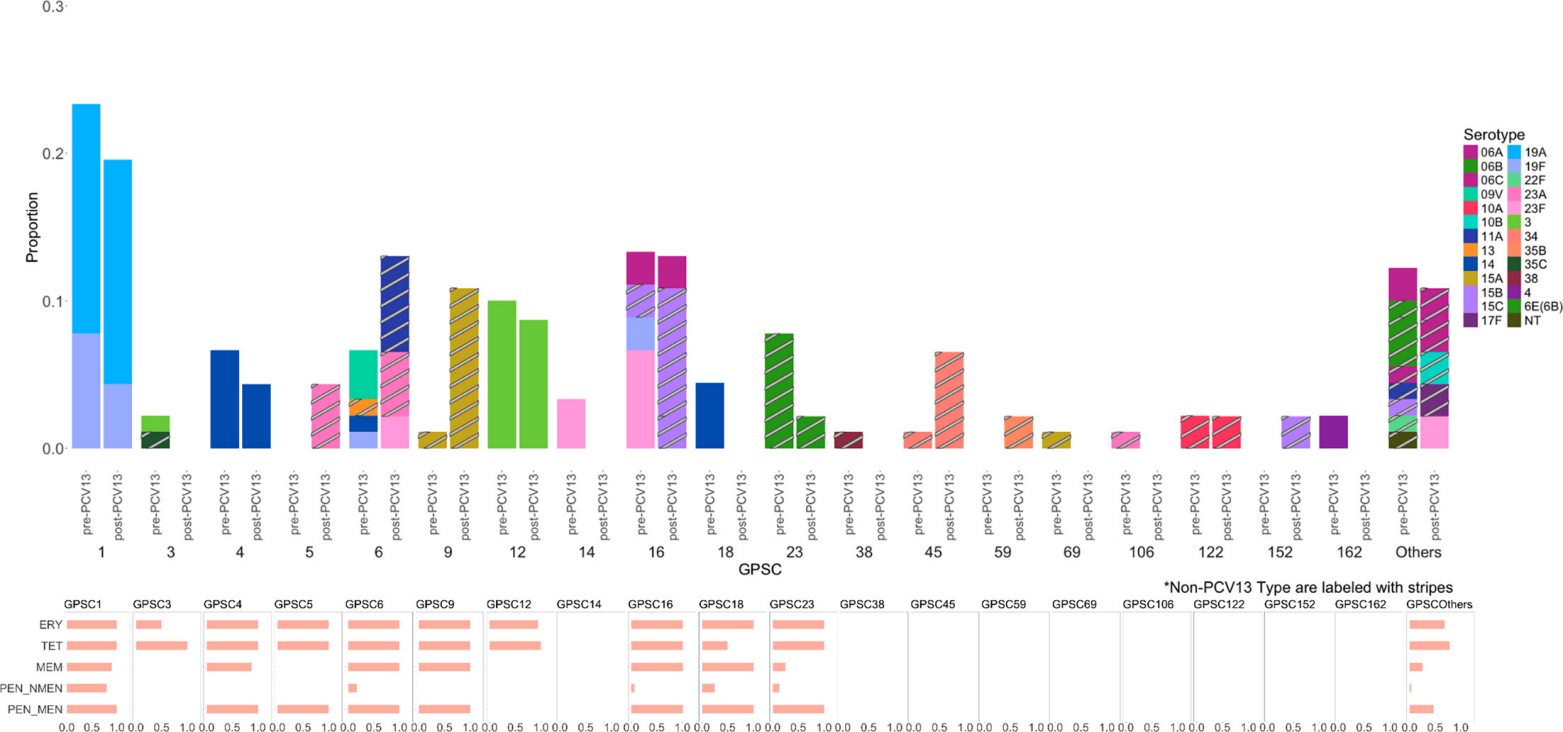
GPSC, serotype and antimicrobial non-susceptible profile of 137 IPD isolates in pre- and post-PCV13 period. (Upper) The GPSC proportion distribution categorized by the PCV13 era. Serotype composition in each GPSC is indicated by colours, and stripes indicate non-PCV13 VT. (Lower) Antimicrobial non-susceptible percentage profile with five antibiotic classes in each GPSC in Taiwan. ERY, erythromycin; TET, tetracycline; MEM, meropenem; PEN_NMEN, penicillin non-meningitis breakpoint; PEN_MEN, penicillin meningitis breakpoint.

Table 2 and Fig. 3 summarize the distribution of GPSCs, MLSTs and serotypes among all 137 invasive isolates that underwent WGS. Seven major GPSCs, including GPSC1, GPSC16, GPSC12, GPSC6, GPSC4, GPSC23 and GPSC9, were each represented by more than 5 isolates and together accounted for 97 of the 137 isolates (70.8%). These dominant GPSCs exhibited a wide range of serotypes and sequence types. The remaining 40 isolates were distributed across 19 less common GPSCs.

**Table 2.** Characteristics of invasive pneumococcus (*n*=137) in Taiwan, 2006–2022 (extracted from Table S1)*

GPSC (No.)∗	Pre-PCV (*n*=91)	Post-PCV (*n*=46)	MLST	PCV13 serotype coverage rate, no. (%)	MDR‡ no. (%)
GPSC1 (30)	**19A** (14), **19F** (7)	**19A** (7), **19F** (2)	ST320 (20), ST236 (4), ST257 (2), ST271 (2), ST1645 (1), ST8521 (1)	30 (100)	30 (100)
GPSC16 (18)	**6A** (2), 15B (2), **19F** (2), **23F** (6)	**6A** (1), 15B (4), 15C (1)	ST81 (10), ST83 (7), nontypeable (1)	11 (61.1)	18 (100)
GPSC12 (15)	**3** (11)	**3** (4)	ST180 (13), ST2570 (1), nontypeable (1)	15 (100)	14 (93.3)
GPSC6 (12)	13 (1), **14** (1), **9V** (3), **19F** (1)	11A (3), 23A (2), **23F** (1)	ST166 (11), ST1569 (1)	8 (66.7)	12 (100)
GPSC4 (8)	**14** (6)	**14** (2)	ST876 (8)	8 (100)	8 (100)
GPSC23 (8)	**6B** (7)	**6B** (1)	ST90 (4), ST95 (3), nontypeable (1)	8 (100)	8 (100)
GPSC9 (6)	15A (1)	15A (5)	ST63 (6)	0 (0)	6 (100)
Other (40)†				20 (50)	26 (65)
Total (137)	42 serotypes (128), nontypeable (9)			98 (71.5)	122 (89.1)

*Only GPSCs over five isolates were listed here.

†Others included GPSC76 (4), GPSC18 (4), GPSC45 (4), GPSC14 (3), GPSC122 (3), GPSC162 (2), GPSC3 (2), GPSC5 (2), GPSC321 (1), GPSC59 (1), GPSC69 (1), GPSC172 (1), GPSC106 (1), GPSC38 (1), GPSC244 (1), GPSC73 (1), GPSC152 (1), GPSC391 (1), GPSC554 (1) and five non-clustered isolates.

‡MDR, including resistant to ≥3 classes of antimicrobials according to the *in silico* antimicrobial resistance results.

Serotypes in bold indicate vaccine serotypes covered by PCV13.

Among the 26 GPSCs identified, 11 exclusively expressed VTs, while 12 were associated solely with NVTs. Although the overall prevalence of major GPSCs remained relatively stable between the pre- and post-PCV13 periods, several GPSCs carrying NVTs emerged following the introduction of PCV13. These included GPSC5, GPSC6, GPSC9, GPSC45 and GPSC152 (Fig. 3). Notably, GPSC9 showed a significant post-PCV13 increase (*P*=0.016; Table S3) and was exclusively composed of serotype 15A/ST63 isolates.

GPSC1 was the most prevalent lineage and was primarily composed of serotype 19A/ST320 isolates (19/29, 65.5%). GPSC16 showed a shift toward NVT 15B/C, increasing from 16.7% pre-PCV13 to 83.3% post-PCV13, while GPSC6 transitioned from mainly VT serotypes (14, 9V and 19F) to NVTs such as 11A and 23A (Table 2).

The predicted MIC values for penicillin from WGS were all within ±1 log2 dilution compared with those obtained by BMD. Overall concordance between phenotypic and *in silico* resistance was ≥94% for all tested agents (Table S5). Given this high agreement, *in silico* predictions were used for subsequent analyses. Among the 137 invasive isolates, 97.1% were non-susceptible to at least one antibiotic, and 89.1% met the criteria for MDR (Table 2).

Penicillin resistance (MIC >0.12 mg l^−1^) was primarily linked to two *pbp1a:2b:2x* mutation profiles*,* 15 : 12 : 18 (20/101) and 13 : 11 : 16 (19/101), accounting for 19.8% and 18.8% of resistant isolates, respectively (Table S6). The *pbp 1 a:2b:2x* (15 : 12 : 18) profile was mainly found in GPSC6/ST166 (*n*=6) and GPSC16/ST81 or ST83 (*n*=14) and was associated with intermediate meropenem MICs (0.5 mg l^−1^) while retaining susceptibility to amoxicillin/clavulanate. In contrast, the *pbp* 1 a:2b:2x (13 : 11 : 16) profile was linked to GPSC1/serotype 19A/ST320 and conferred resistance to amoxicillin/clavulanate (MIC=8 mg l^−1^). Both mutation profiles were present in isolates from both pre- and post-PCV13 periods.

Of the 137 isolates, 128 (93.4%) carried the *tetM* gene associated with tetracycline resistance. Erythromycin resistance genes were detected in 125 isolates, including *ermB* (*n*=105), *mefA* (*n*=19) and *mefE* (*n*=1). Mutations conferring fluoroquinolone resistance were identified in *gyrA* (*n*=9), *parC* (*n*=5) and *parE* (*n*=1). In terms of virulence factors, *pili1* and *pili2* genes were present in 43 and 30 isolates, respectively, with *pili1* predominantly found in GPSC1 (*n*=25). Notably, all *pili2*-positive isolates belonged to GPSC1.

### Phylogenetic structure and lineage diversification in Taiwan

**Fig. 4.**
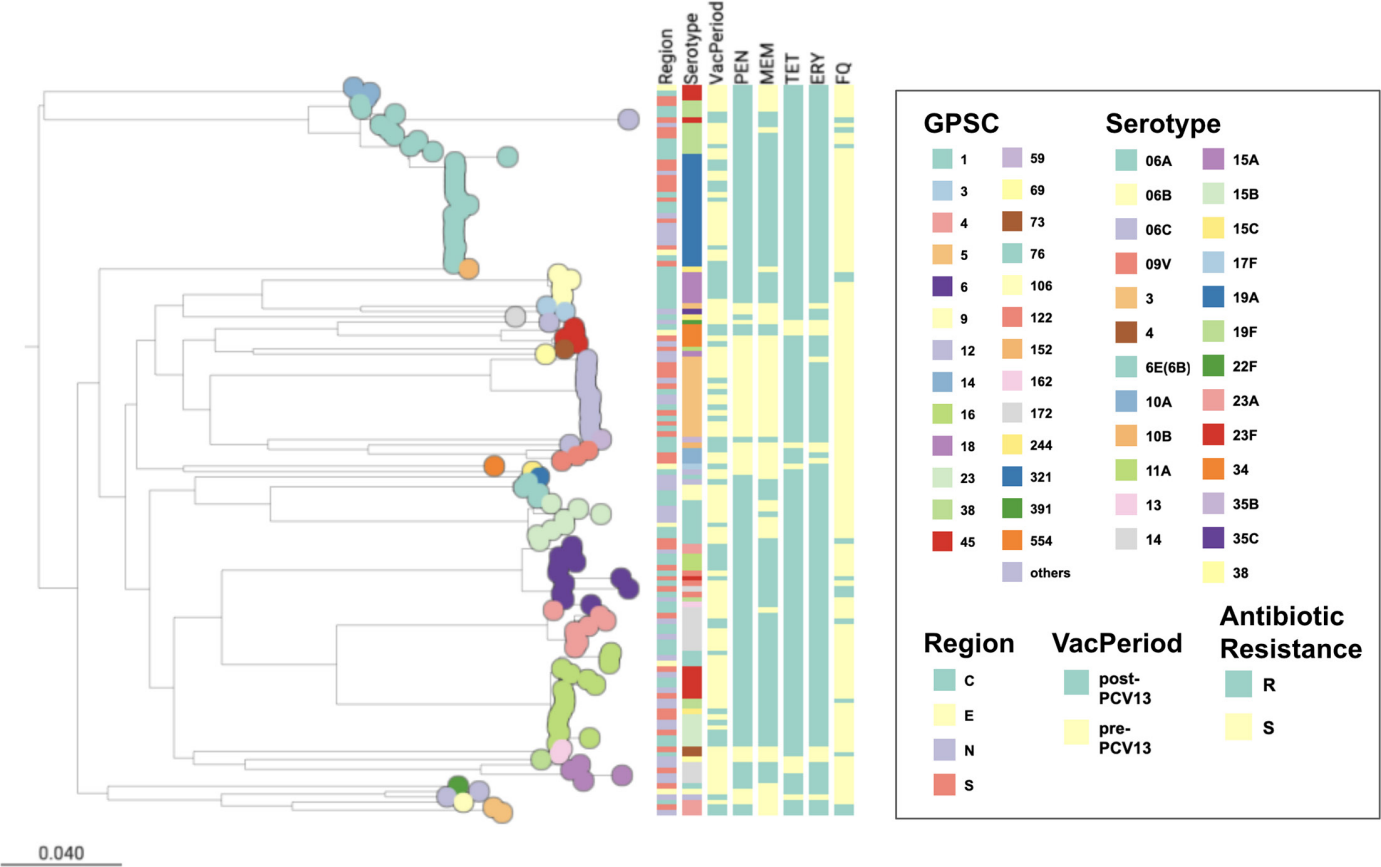
The phylogeny of the 137 IPD isolates collected in Taiwan. A maximum likelihood phylogeny of the 137 IPD isolates is shown on the left, and the scale of nt substitution is shown at the bottom left. The leaf nodes are coloured by GPSC. Region, serotype, vaccine period and antibiotic resistance profile are shown in the coloured columns. Regions in Taiwan are divided into Central (C), East (E), North (N) and South (S). Antimicrobial susceptibility is classified as resistant (R) and susceptible (S).

The phylogenetic tree based on the whole genome of these 137 Taiwan *S. pneumoniae* isolates revealed substantial genetic diversity (Fig. 4, https://microreact.org/project/rzJGdFK6MBWANcfLy1K3rH-taiwan-137-isolates).

GPSC1 formed a tightly clustered lineage dominated by the serotype 19A/ST320 clone, which was observed in both pre- and post-PCV13 periods. These isolates carried the *pbp 1 a:2b:2x* (13 : 11 : 16) resistance profile and were widely distributed across all four regions of Taiwan, indicating sustained and geographically widespread clonal expansion.

In contrast, GPSC6 demonstrated extensive serotype diversification after PCV13 use. To understand the change in serotype composition within GPSC6, we combined GPSC6 isolates from the TSAR collection and GPSC6 isolates from the Global Pneumococcal Sequencing (GPS) dataset and created a recombination-corrected phylogeny (https://microreact.org/project/215hf5ztUDPYsPqYzRNNDt-gpsc6taiwangpsphylogeny). The frequent capsular switching in GPSC6 is particularly distinct in Taiwan compared with other countries. While GPSC6 isolates from Taiwan exhibit various serotypes, GPSC6 isolates from other countries show one or few serotypes. For example, isolates collected from Cambodia consistently show serotype 11A.

**Fig. 5.**
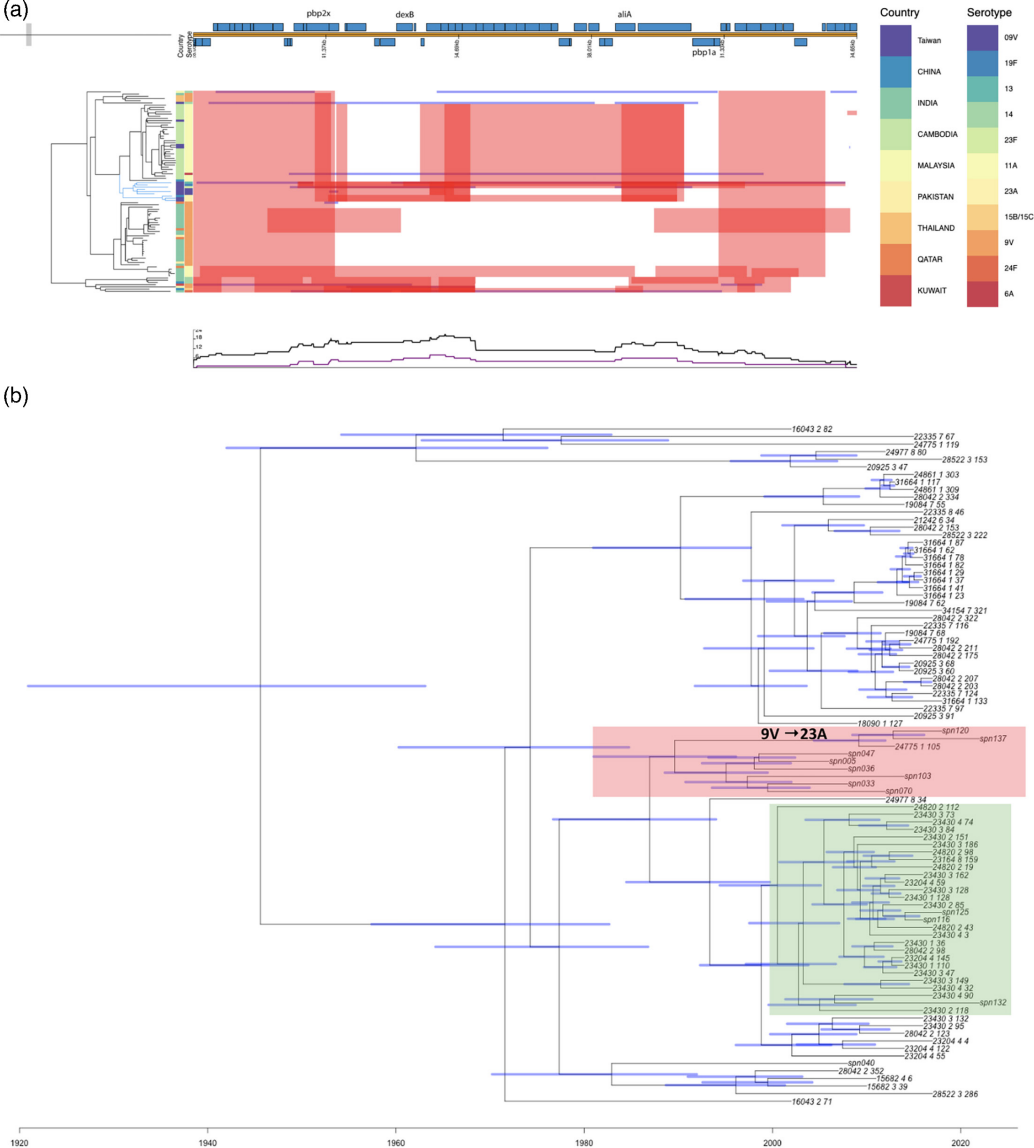
The phylogenetic relationship of GPSC6 pneumococcal isolates collected in Taiwan from the TSAR collection and other Asian countries in the GPS dataset. (**a**) Recombination-free phylogeny and recombination sites of GPSC6 isolates. The upper panel indicates the gene position and annotation, and the plot focuses on the cps region. The middle panel shows the recombination blocks where red blocks indicate the recombination shared by the corresponding clade and blue blocks indicate the recombination that is unique to the corresponding isolate. In the recombination-free phylogeny, a clade containing eight Taiwan isolates is highlighted in blue. The lower panel shows the recombination frequency in the presented region, where the black line indicates overall recombinations across all GPSC6 samples in this collection, while the purple line indicates recombination found in the blue clade. Country and serotype information is shown in the coloured columns. (**b**) A time-resolved phylogeny of the GPSC6 isolates. The time scale is indicated in the x-axis. The blue bars show the 95% credible interval of the inferred time of the inner nodes. Two clades including isolates from the TSAR collection are coloured in red and green. A branch corresponding to a capsular switching event (serotype 9V to 23A) is indicated.

To explore the relationship further, we constructed GPSC6-specific recombination-corrected phylogenetic trees on isolates collected from Asian countries and identified recombination blocks in Taiwan GPSC6 isolates (Fig. 5a). Increasing recombination frequency was found among *cps* locus in Taiwan GPSC6 isolates. Two Taiwan isolates, spn70 (serotype 13, collected in 2012) and spn103 (serotype 23F, collected in 2014), showed exceptionally high recombination frequency (r/m=15.74 and 21.96, respectively) compared with other GPSC6 isolates in other countries (Fig. 5a), and both spn70 and spn103 carry unique recombination blocks, leading to capsular switching (blue blocks in Fig. 5a). spn137 and spn120 also carry a recombination block including 86 SNPs, leading to the serotype change to 23A. To investigate when the recombination causing the serotype switch happened, we generated a time-resolved phylogeny by BactDating. Isolates collected from Taiwan were split into two different clades. The majority of Taiwan isolates were clustered together with the most recent common ancestor (MRCA) in the early 1990s (1988–1997, 95% credible interval, clade I coloured in red in Fig. 5b). Aforementioned spn70, spn103 and spn33 were all situated in this clade, indicating that the frequent recombination events might have occurred within Taiwan (Fig. 5b). A serotype 9V (spn047) to 23A (spn120 and spn137) capsular switching event was identified on a branch spanning the vaccine introduction period. Clade II (coloured in green in Fig. 5b) includes spn125, spn116 and spn132, which form a cluster with isolates from Cambodia with MRCA in the early 2000s (2003–2009, 95% credible interval). Together, these results suggest that the diverse genetic background in GPSC6 isolates from Taiwan may result from transmission between countries and frequent recombination events after being introduced to Taiwan.

We also investigated the NVT-expanding GPSC16 isolates in Taiwan by combining them with the GPSC16 isolates collected from the GPS dataset and constructed a phylogeny (https://microreact.org/project/qUbfafFwwWoqow1omh38xN-gpsc16taiwangpsphylogeny). The phylogeny showed that most GPSC16 isolates in Taiwan were phylogenetically clustered together while continuously evolving and the most recent ones carrying serotype 15B. This indicates that the pre-existing GPSC16/15B is expanding within the GPSC16 lineage in Taiwan (Fig. S2).

## Discussion

This study represents the first comprehensive analysis of the molecular epidemiology of invasive pneumococcal isolates from Taiwanese individuals. Our findings indicate that GPSC6, GPSC16 and GPSC9 are the predominant lineages consisting of non-PCV13 serotypes. Notably, GPSC6 exhibits a high frequency of recombination events following the introduction of PCV13. This capacity for rapid capsular switching highlights its adaptability under vaccine pressure, posing significant implications for pneumococcal disease management and vaccine strategies in Taiwan.

The introduction of PCV13 substantially reduced pneumococcal infections across all age groups. However, a shift in disease burden toward the elderly was observed, with an increased proportion of IPD in this population. This pattern has also been reported in both Taiwan and the USA [26,27]. In our study, serotypes 19A, 19F and 6A remained common among adults and the elderly despite inclusion in PCV13, likely due to limited indirect protection from childhood vaccination. Contributing factors may include the absence of routine pneumococcal vaccination for adults aged 19–64 years and the lower immunogenicity of PPSV23, which has been used for those aged 65 years and older [28]. In 2023, Taiwan began co-administering PCV13 and PPSV23 to individuals aged 65 years or older to enhance protection. These findings underscore the importance of ongoing surveillance to evaluate the impact of expanded vaccination strategies in older populations.

In our study, GPSC1 is predominantly associated with serotypes 19A and 19F and remains the most significant lineage causing invasive syndromes with the highest prevalence of antimicrobial resistance, even after the introduction of PCV13. We found that the IPD strains in the post-PCV13 period were mainly found in adults with serotype 19A. In previous studies, many countries have experienced an increase in serotype 19A prevalence after shifting from PCV13 to PCV10 due to different serotype coverage [29,30]. However, PCV13 has been consistently used since 2015 in Taiwan without change, yet the high prevalence of serotype 19A has persisted despite its use. This persistence may be attributed to two main factors: vaccine escape and antimicrobial selective pressure. A variant of serotype 19A/CC320 is responsible for ~10% of IPD in Ireland and has been linked to PCV13 vaccine failures and breakthroughs [31]. This study also demonstrated that vaccine failure or breakthrough cases were more frequently associated with GPSC1-CC320 than with other GPSCs [31]. These findings highlight the urgent need for enhanced strategies to address the challenges posed by GPSC1-CC320 and mitigate its impact on public health.

Furthermore, Taiwan has had extensive public health insurance coverage since 1999, and the consumption of antimicrobials has increased year by year [32]. The selective pressure from widespread antimicrobial use may be a contributing factor to the persistent high prevalence of serotype 19A in Taiwan, particularly due to the MDR harboured by GPSC1/19A isolates. The resistome identified in these strains, characterized by specific mutations in PBPs, *pbp1a, 2b, 2x* (13 : 11 : 16), indicates a regional trend of antibiotic resistance that poses significant treatment challenges. The persistence of GPSC1 in Taiwan underscores its ability to adapt to both antimicrobial and vaccine selection pressures, raising concerns about its potential continued spread.

Changes in serotype prevalence among pneumococcal populations are driven by both serotype replacement and capsular switching [33]. Specifically, NVTs within GPSC16 began to replace VTs following the introduction of PCV13, providing a clear example of serotype replacement. In contrast, GPSC6 demonstrated frequent recombination events, particularly within the *cps* locus, as revealed by recombination analysis. These recombination events, involving lineages from countries such as Cambodia, may be attributed to the substantial influx of foreign migrant workers and international marriages in Taiwan since 1989. The elevated recombination rate in GPSC6 likely represents an adaptive mechanism, enabling it to quickly respond to vaccine-induced selective pressures and sustain its prevalence. The identification of the previously unreported ST166 isolates within GPSC6 offers new insights into pneumococcal evolution in Taiwan, potentially representing a distinct evolutionary pathway that requires further investigation. We examined Spain^9V^/23A/ST166 strains from a Taiwanese cohort collected between 2019 and 2021 to determine their GPSC classification, finding that they also belong to GPSC6 [34]. These 23A/ST166 isolates also exhibited pbp*1a: 2b: 2x* mutations (15 : 11 : 299), identical to those observed in our isolates, spn120 and spn137. However, our study was not restricted to 23A/ST166 strains, and we additionally observed numerous instances of capsular switching. The emergence of NVTs dominated by GPSC6 and GPSC16 is particularly concerning, as both lineages harbour PBP mutations, rendering them non-susceptible to penicillin and meropenem. This underscores the need for increased attention and further research on these lineages due to their potential public health impact.

In this study, the pneumococcal invasiveness rate among the elderly increased significantly, from 7.6% in 2006 to 27.7% in 2022. Subgroup analysis revealed that, in the post-PCV13 period, IPD strains in the elderly were predominantly NVTs, particularly 15A. The emergence of the non-PCV13 vaccine covering GPSC9/15A is highly concerning, not only in Taiwan but also globally [35]. Six years after PCV13 implementation, serotype 15A has become the most prevalent NVT in Mongolia, especially among infants and toddlers [36]. In Canada, the increasing resistance of the GPSC9/15A strain to multiple antibiotics, including macrolides, is particularly worrisome due to its rapid spread and high clonality [37]. Moreover, this strain’s ability to switch to an unencapsulated phenotype by inserting the pspC virulence factor into the *cps* locus further underscores its potential virulence and the significant challenges it poses to public health [38].

Our study is limited by the relatively small number of invasive pneumococcal isolates subjected to WGS, which may under-represent the genetic diversity of pneumococcus in Taiwan. The number of isolates collected in 2020 was also lower than expected, likely due to the impact of non-pharmaceutical interventions during the coronavirus disease 2019 pandemic, which may have influenced the overall incidence of pneumococcal disease [39]. Despite these limitations, this study remains the largest investigation of invasive pneumococcal strains in Taiwan and provides valuable insights into the molecular epidemiology of *S. pneumoniae* across different age groups.

## Conclusion

Since PCV13 introduction, pneumococcal populations in Taiwan have undergone significant changes, driven by serotype replacement in GPSC16 and capsular switching in GPSC6, resulting in reduced VT strains. However, the continued spread of serotype 19A/ST320 and the emergence of multidrug-resistant GPSC9 highlight the adaptability of *S. pneumoniae* to vaccine and antimicrobial pressures. The shift in IPD burden toward the elderly necessitates close monitoring, particularly following changes in the PCV13 vaccination programme. These results emphasize the urgent need to incorporate both serotype surveillance and genomic monitoring into routine public health policy planning, particularly for high-risk age groups.

## Supplementary material

10.1099/mgen.0.001498 Supplementary Material 1.

## Abbreviations

AMR: antimicrobial resistance
BMD: broth microdilution
CLSI: Clinical and Laboratory Standards Institute
GPSC: Global Pneumococcal Sequence Cluster
ICU: intensive care unit
IPD: invasive pneumococcal disease
MDR: multidrug resistance
MIC: minimum inhibitory concentration
MLST: multilocus sequence typing
MRCA: most recent common ancestor
NVT: non-vaccine serotype
PBP: penicillin-binding protein
PCV: pneumococcal conjugate vaccine
QC: quality control
TSAR: Taiwan Surveillance of Antimicrobial Resistance
VT: vaccine type
WGS: whole-genome sequencing
